# Tetraspanins and Transmembrane Adaptor Proteins As Plasma Membrane Organizers—Mast Cell Case

**DOI:** 10.3389/fcell.2016.00043

**Published:** 2016-05-10

**Authors:** Ivana Halova, Petr Draber

**Affiliations:** Department of Signal Transduction, Institute of Molecular Genetics, Academy of Sciences of the Czech RepublicPrague, Czech Republic

**Keywords:** CD9, LAT, NTAL, IgE receptor, plasma membrane, membrane microdomains, signal transduction

## Abstract

The plasma membrane contains diverse and specialized membrane domains, which include tetraspanin-enriched domains (TEMs) and transmembrane adaptor protein (TRAP)-enriched domains. Recent biophysical, microscopic, and functional studies indicated that TEMs and TRAP-enriched domains are involved in compartmentalization of physicochemical events of such important processes as immunoreceptor signal transduction and chemotaxis. Moreover, there is evidence of a cross-talk between TEMs and TRAP-enriched domains. In this review we discuss the presence and function of such domains and their crosstalk using mast cells as a model. The combined data based on analysis of selected mast cell-expressed tetraspanins [cluster of differentiation (CD)9, CD53, CD63, CD81, CD151)] or TRAPs [linker for activation of T cells (LAT), non-T cell activation linker (NTAL), and phosphoprotein associated with glycosphingolipid-enriched membrane microdomains (PAG)] using knockout mice or specific antibodies point to a diversity within these two families and bring evidence of the important roles of these molecules in signaling events. An example of this diversity is physical separation of two TRAPs, LAT and NTAL, which are in many aspects similar but show plasma membrane location in different microdomains in both non-activated and activated cells. Although our understanding of TEMs and TRAP-enriched domains is far from complete, pharmaceutical applications of the knowledge about these domains are under way.

## Introduction

The plasma membrane, as well as other cellular membranes, is a very complex structure composed of a plethora of proteins and a variety of lipids organized into two asymmetrical leaflets. The proper function of cellular membranes depends on the composition of the leaflets and intermolecular communication of the membrane components. This is especially important in such complex processes as signal transduction from plasma membrane receptors into the cytoplasm and nucleus. Cellular membranes play a key role in signal transduction in both directions, inside-out and outside-in. For years it has been thought that the lipid composition of the membrane allows formation of signaling platforms that are critical for membrane functioning and that membrane lipids and proteins play key roles as membrane organizers (Singer and Nicolson, 1972; van Meer et al., 2008; Simons and Gerl, 2010; Treanor and Batista, 2010; Simons and Sampaio, 2011).

Many systems have been used to study the structure-function relationships of the plasma membrane components. Among them are mast cells (MCs) or MC lines. MCs are effector cells of the immune system that are able to react to external stimuli by rapid release of numerous allergy mediators from cytoplasmic granules and/or by production and secretion of a variety of cytokines and chemokines. Activation of MCs is initiated by binding of ligands [e.g., antigens (Ags)] to plasma membrane receptors or their complexes [e.g., immunoglobulin (Ig)E bound to high-affinity IgE receptor (FcεRI)]. This binding initiates cell activation events, which involve a number of signal transduction molecules forming functionally and spatially connected units, called signalosomes. Receptor-mediated responses vary in strength and duration. For example, aggregation of FcεRIs by multimeric Ag-IgE complexes leads to phosphorylation of several proteins, followed by Ca^2+^ response, in seconds after triggering, release of pre-formed granules containing various mediators, in minutes after activation, and ending by *de novo* synthesis of lipid mediators, cytokines and chemokines in tens of minutes and hours after activation (reviewed in Marshall, 2004; Galli et al., 2008; Kalesnikoff and Galli, 2008; Galli and Tsai, 2012). These signaling events could have dramatic physiological consequences, such as causing allergy disease (Galli and Tsai, 2012) or breaking down poison in snake venom (Metz et al., 2006), and therefore must be precisely regulated (Galli, 2016). There are numerous signal transduction regulators localized in the plasma membrane, membrane proximal, in the endoplasmic reticulum, as well as in the cytoplasm. This review is focused on two groups of transmembrane proteins that are involved in plasma membrane receptor regulation and functioning. The first group consists of tetraspanins, which span the plasma membrane four times and form two extracellular domains and short intracellular tails. The second group consists of transmembrane adaptor proteins (TRAPs), which possess a short extracellular domain, one transmembrane domain and a long intracellular tail with several tyrosines that once phosphorylated can serve as anchor for different proteins and in this way influence signal transduction. Although these two groups of proteins are structurally different, they share some common properties, such as palmitoylation and ability to interact with a plethora of plasma membrane-bound or intracellular proteins. Furthermore, recent studies with MCs found a cross-talk between proteins in these two groups in the regulation of plasma membrane-bound signaling events (Hálová et al., 2013).

## Tetraspanins

**Figure 1:**
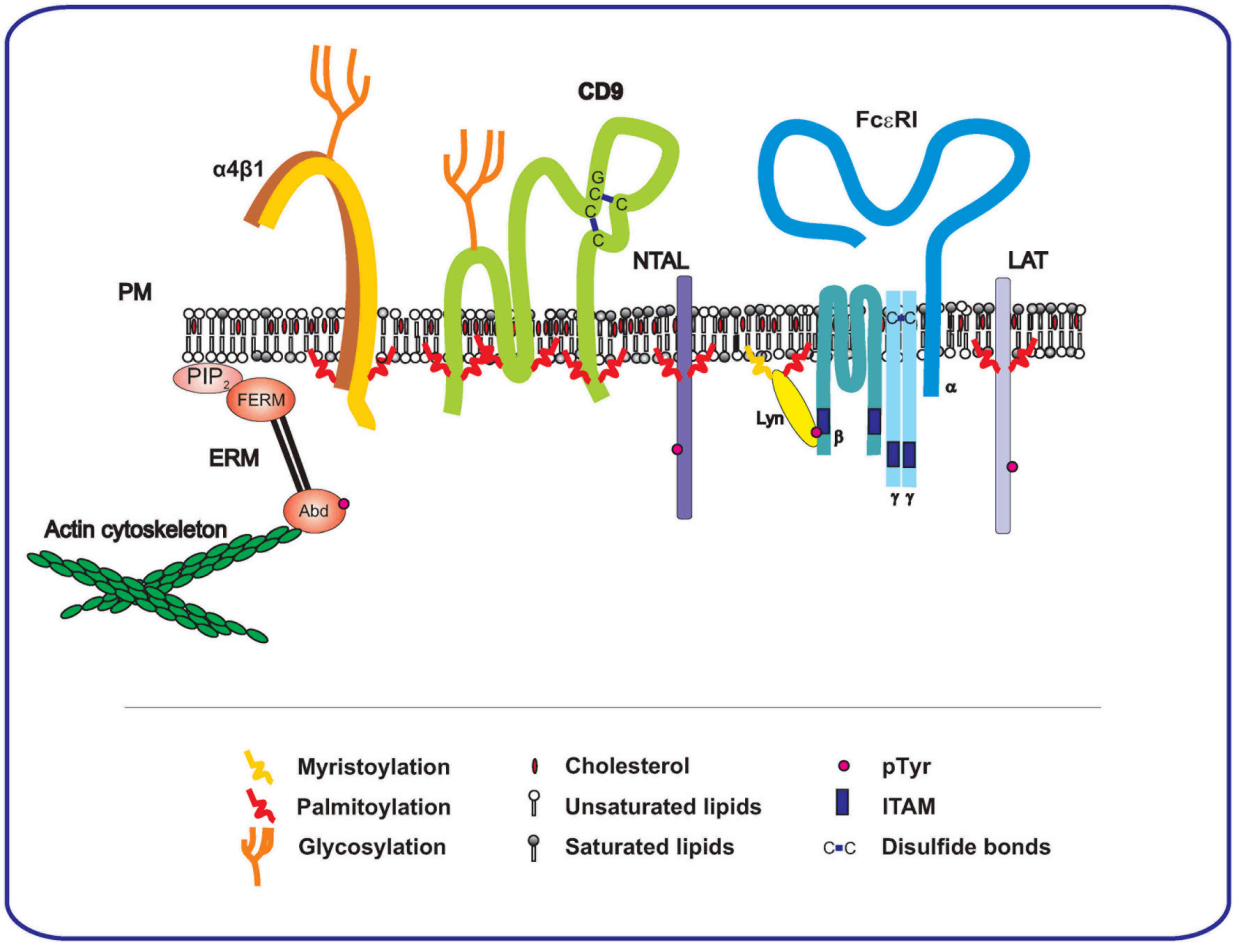
**Involvement of TEMs and TRAP-enriched domains in FcεRI signalosome**. At the level of the plasma membrane, there is a cross-talk of FcεRI, consisting of four transmembrane subunits (α,β, and γγ), with tetraspanins and TRAPs. These interactions affect FcεRI signal transduction. Phosphorylated ITAMs of FcεRI bind LYN kinase, which is connected to the membrane by palmitoylation and myristoylation. Two important TRAPs, NTAL and LAT, which occupy different nanodomains, are also palmitoylated. Tetraspanins are represented by CD9, which contains six palmitoylation domains and one possible glycosylation site in a small intracellular loop, two disulfide bonds and tetraspanin conserved motif CCG are also depicted in the figure. Finally, integrins are represented by palmitoylated α4β1 that is located in close proximity of CD9. The membrane is connected with the actin cytoskeleton through the FERM (4.1,ezrin/radixin/moesin) domain and actin-binding domain (Abd) of ERM proteins. FERM binds directly to phosphatidylinositol 4 5-bisphosphate (PIP2).

Tetraspanins are an evolutionarily conserved superfamily of transmembrane proteins with characteristic features. They have four transmembrane domains and two extracellular loops, a small one (SEL) and a large one (LEL). LEL possesses a conserved CCG motif and at least two other cysteine residues that form disulfide bonds inside the LEL domain (Figure 1). There are two main post-translational modifications occurring in tetraspanins. Most of them possess one or more N-glycosylation sites at the LEL domain with two exceptions, cluster of differentiation (CD)9, which possesses an N-glycosylation site in SEL, and CD81, which is non-glycosylated (Boucheix and Rubinstein, 2001). Interestingly, all of the so far studied tetraspanins contain a palmitoylation site (Charrin et al., 2002; Yang et al., 2002, 2004). Palmitoylation is a modification that tetraspanins share with a plethora of other transmembrane or membrane-associated proteins, such as TRAPs [including linker for activation of T cells (LAT), non-T cell activation linker (NTAL; also called LAT2), and phosphoprotein associated with glycosphingolipid-enriched membrane microdomains (PAG; also called CSK-binding proteins)] (Draber et al., 2011a; Stepanek et al., 2014), integrins (Berditchevski, 2001; Gagnoux-Palacios et al., 2003; Yang et al., 2004), SRC kinases (Kovářová et al., 2001; Gilfillan and Rivera, 2009), and others. Examples of palmitoylated proteins expressed on the MC membrane are presented in Figure 1. Palmitoylation is important for the protein topography and its functioning in the plasma membrane (see below).

Tetraspanins are known regulators of cell migration (Boucheix and Rubinstein, 2001) and are involved in tumor progression and metastasis (Zöller, 2009). Some tetraspanins such as CD82, and often also CD9, are downregulated in advanced stages of cancer; their absence is a sign of poor prognosis in patients with several types of cancer. These tetraspanins are considered as tumor suppressors. However, some other tetraspanins, such as CD151 and TSPAN8, have been found upregulated in metastases and their upregulation was associated with poor prognosis, suggesting that they serve as tumor promotors (reviewed in Zöller, 2009). The authors speculated that the different roles of tetraspanins in tumor progression could be the result of specific tetraspanin abundance in exosomes. This could positively or negatively influence the fusion of exosomes with plasma membranes in partner cells, depending on the tetraspanin type. In this way exosomes possessing different tetraspanins could exhibit different delivery of important communicators such as mRNA and microRNA and also proteins important in cell-cell communication (Zöller, 2009).

Several tetraspanins display broad tissue expression (e.g., CD9, CD81, CD151), whereas others are restricted e.g., to leukocytes (CD37, CD53). Only a few tetraspanins (UP1a, UP1b, peripherin and ROM-1) have their distribution limited to specific tissue (Tarrant et al., 2003; Charrin et al., 2009, 2014). Although tetraspanins are abundant proteins in many cell types, animals deficient in selected tetraspanins usually do not exhibit striking phenotypes, probably due to the redundancy and functional compensation of individual tetraspanins. On the other hand, mutations in individual tetraspanin genes have been described as a cause of several life-threatening diseases (Kajiwara et al., 1993, 1994; Zemni et al., 2000; Karamatic et al., 2004; van Zelm et al., 2010). In some cases, mice lacking a particular tetraspanin mimicked the phenotype observed in humans with a defect in the same tetraspanin (Hemler, 2005).

Although several tetraspanins (CD9, CD37, CD53, CD63, CD81, CD82, CD151) are abundantly expressed on the plasma membrane of MCs and/or secretory vesicles (Table 1), their role in MC physiology and activation events is not completely understood. The problem is that for some tetraspanins there are no commercially available antibodies and/or knockout (KO) animals for such studies. The role of tetraspanins in MCs was reviewed in 2012 (Köberle et al., 2012) and since then, several new studies have appeared highlighting the significance of these membrane organizers in MC physiology. The novel findings are summarized below.

**Table 1:** **Selected tetraspanins expressed in mast cells and their function**.

**Gene symbol**	**Most used aliases**	**KO**	**Phenotype of KO mice, general/in mast cells**	**Other important functions in mast cells**
CD9	BTCC-1, DRAP-27, MIC3, MRP-1, TSPAN-29, TSPAN29	Le Naour et al., 2000	Deficiency in sperm-egg fusion (Kaji et al., 2000; Le Naour et al., 2000; Miyado et al., 2000; Wright et al., 2004)/not studied	Antibodies against CD9 block MCs migration toward IL-16 (Qi et al., 2006) and Ag (Hálová et al., 2013) and induce Ca^2+^ release and phosphorylation of several substrates (Hálová et al., 2013)
CD37	GP52-40, TSPAN26	Knobeloch et al., 2000	Mild alteration of immune system (Knobeloch et al., 2000)/normal levels of IgE, MCs not particularly studied (Knobeloch et al., 2000)	
CD53	MOX44, Ox-44, TSPAN25			Associated with population asthma risk but not directly connected to MCs (Lee et al., 2013)
CD63	LAMP-3, ME491, MLA1, OMA81H, TSPAN30	Schröder et al., 2009	Mild defects, altered water balance (Schröder et al., 2009)/reduced degranulation, TNF-α secretion, and PCA (Kraft et al., 2013)	Antibody against CD63 suppresses degranulation and PCA (Kraft et al., 2005)
CD81	TAPA-1, CVID6, S5.7, TSPAN28	Maecker and Levy, 1997	Female infertility (Rubinstein et al., 2006), nervous system malfunctions (Geisert et al., 2002), reduced IL-4 production (Maecker et al., 1998), lower expression of CD19 on B cells (Maecker and Levy, 1997)/not studied	Antibody against CD81 suppresses degranulation and PCA (Fleming et al., 1997)
CD82	KAI1 4F9, C33, GR15, IA4, R2, SAR2, ST6, TSPAN27	Risinger et al., 2014	Mild changes in phenotype, could lead to changes in early establishment of proliferation and division when challenged with a new environment (Risinger et al., 2014)/not studied	
CD151	GP27, MER2, PETA-3, RAPH, SFA1, TSPAN24	Wright et al., 2004; Sachs et al., 2006	Bleeding (Wright et al., 2004), decreased angiogenesis (Takeda et al., 2007), kidney failure (Sachs et al., 2006)/increased late phase of PCA and production of proinflammatory cytokines (Abdala-Valencia et al., 2015)	CD151 is upregulated in MCs upon FcεRI activation (Abdala-Valencia et al., 2015)

## CD9

**Figure 2:**
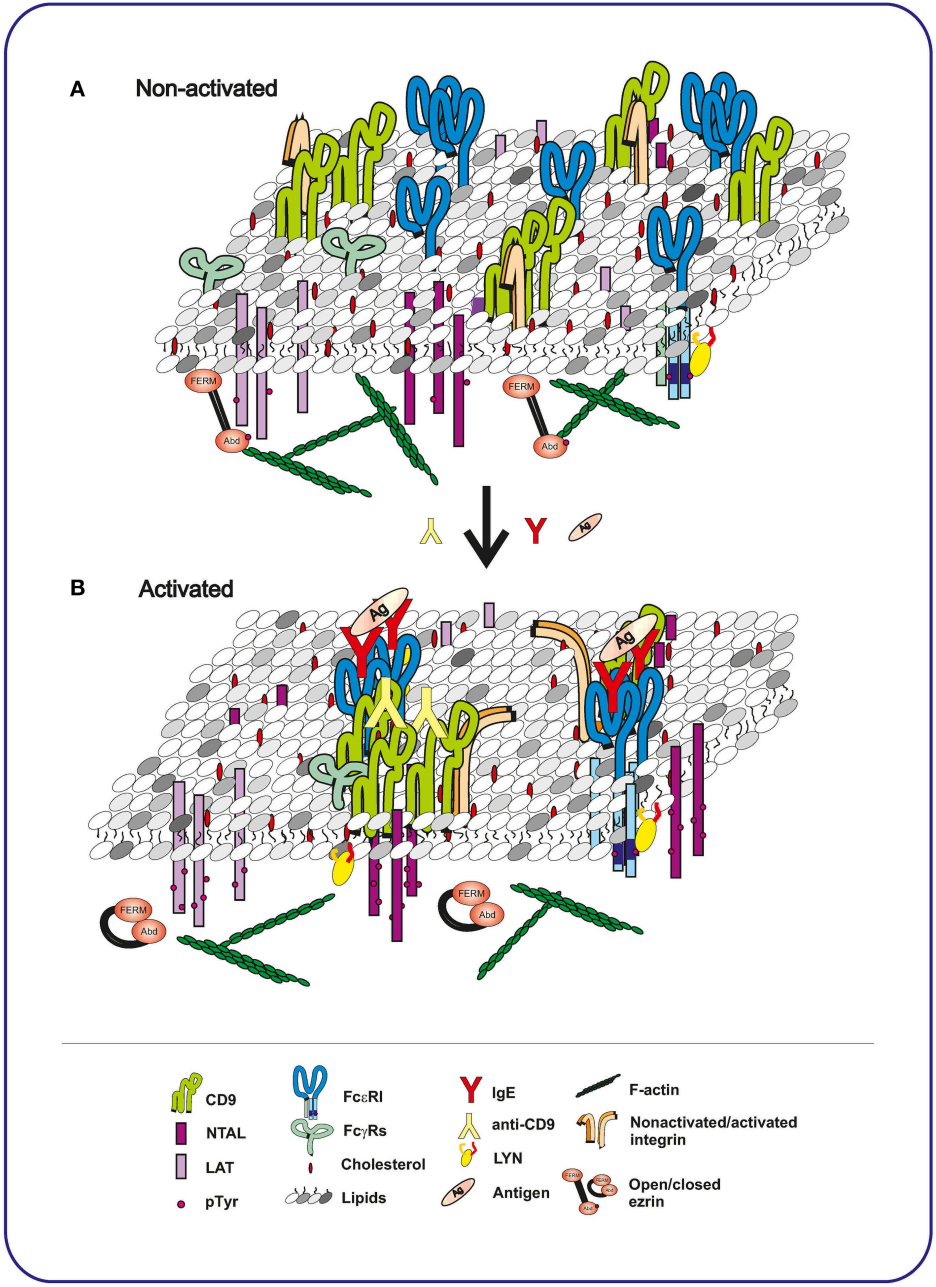
**Topography of plasma membrane components in mast cells before and after activation with Ag or CD9-specific antibody. (A)** In non-activated cells, tetraspanin CD9 co-localizes with integrin in TEMs. These domains are topographically different from LAT- or NTAL-containing nanodomains, each occupying a distinct plasma membrane region. FcεRI receptor is associated with LYN kinase in another membrane nanodomains. **(B)** Binding of Ag-specific IgE to FcεRI, followed by exposure to multivalent Ag, causes multimerization of the FcεRI receptors. NTAL and LAT become phosphorylated, but are still separated in different domains and do not co-localize with FcεRI and with each other. In contrast, antibody-mediated aggregation of CD9 brings CD9 into close proximity of NTAL and FcγRs and causes NTAL phosphorylation. Activation through both FcεRI and CD9 leads to dephosphorylation of ERM and dynamic disconnection of the membrane components and actin cytoskeleton. The conclusions indicated above are based on the interpretation of previously published data (Wilson et al., 2002; Volná et al., 2004; Hálová et al., 2013).

Although CD9 KO mice have been prepared (Le Naour et al., 2000), they have not been used for the studies of the role of CD9 in MC physiology. Thus, our knowledge about the role of CD9 in MCs comes mainly from the studies using CD9-specific antibodies. Our recent study described a new CD9-specific monoclonal antibody (mAb), 2H9, which caused mast cell degranulation, Ca^2+^ release and tyrosine phosphorylation of several proteins including TRAP NTAL and dephosphorylation of ezrin/radixin/moesin (ERM) family proteins (Hálová et al., 2013). Phosphorylation of NTAL was brought about by complete antibody but not its F(ab)_2_ fragment, suggesting that the Fc fragment of the antibody is involved. Further studies showed that 2H9 antibody produced NTAL phosphorylation in cooperation with FcγRs through SRC family kinase LYN, and that CD9 and NTAL co-localized in the same membrane microdomains after antibody-induced CD9 aggregation. A previous study showed that antibody-induced aggregation of human CD9 expressed in CD9-negative rat basophilic leukemia (RBL) cells, clone 2H3, also caused degranulation, but its F(ab)_2_ fragment not (Higginbottom et al., 2000). The authors speculated that CD9 and FcεRI form complexes, and therefore crosslinking by anti-CD9 antibody induced activation by a mechanism similar to the one induced by aggregation of FcεRI. However, experiments with bone marrow-derived mast cells (BMMCs) showed that CD9 did not co-localize with FcεRI in non-activated cells, but dimerization of CD9 with bivalent antibody induced movement of CD9 into the close proximity of FcεRI (Figure 2). This co-localization was further strengthened by crosslinking of CD9-anti-CD9 complexes with secondary antibody (Hálová et al., 2013). Surprisingly, unlike anti-CD63 (Kraft et al., 2005) or anti-CD81 (Fleming et al., 1997), as discussed below, anti-CD9 did not modulate Ag-induced degranulation (Hálová et al., 2013).

CD9 together with CD81 have been shown to co-localize with a trimeric variant of FcεRI in human FcεRI^pos^ dendritic cells isolated from the skin of patients with atopic dermatitis. In contrast, only moderate expression of CD9 and CD81 was found on FcεRI^neg^ monocytes (Peng et al., 2011). Concomitant activation by FcεRI and CD9 crosslinking resulted in increased interleukin (IL)-10 production compared to crosslinking of the FcεRI alone. In contrast, co-activation of FcεRI with CD81 or activation by aggregation of CD81 alone or CD9 alone had no effect on IL-10 production (Peng et al., 1997). These data can be taken as another evidence that CD9 and FcεRI are somehow functionally cooperating.

Tetraspanins are well known regulators of chemotaxis and migration in several cell types (Berditchevski, 2001; Boucheix and Rubinstein, 2001). In MCs, IL-16 acts as a potent chemoattractant (Qi et al., 2002). Surprisingly, chemotaxis of MCs toward IL-16 was blocked by anti-CD9 antibody or by reduced expression of CD9 using RNA interference (RNAi) approach. These and other findings led to the suggestion that CD9 acts as an alternate IL-16 receptor (Qi et al., 2006). Studies with 2H9 anti-CD9 antibody showed that intact IgG or its F(ab)_2_ fragment block chemotaxis of MCs toward Ag, whereas Fab fragments had only minimal effects on such chemotaxis. These findings suggest that inhibition of chemotaxis toward Ag is caused by events induced by aggregation of CD9 but does not require co-cross-linking of CD9 with FcγR. Decreased expression of CD9 by lentiviral-induced RNAi CD9 knockdown (KD) did not affect migration toward Ag, suggesting that CD9 is not involved in this process. However, the possibility was not excluded that residual CD9 on the cell surface is involved in the proper Ag-induced migration (Hálová et al., 2013).

## CD63

CD63 was found to be located in the vicinity of FcεRI on RBL-2H3 cells (Kitani et al., 1991). In MCs and basophils, CD63 is expressed at the cell surface and at the membrane of secretory lysosomes, including serotonin-containing granules that during activation fuse with the plasma membrane. Therefore, CD63 is extensively used as an activation marker of basophils. As the response acts as “all-or-nothing” per cell, basophils either do not bind the anti-CD63 mAb at all, or they bind a maximal amount of the mAb, so that activated basophils can be easily identified (Knol et al., 1991; Hoffmann et al., 2015). Unlike basophils, a significant amount of CD63 is also expressed on non-activated human MCs of different types, but similarly to basophils it is also upregulated after activation (Valent et al., 2001). Recently, two structurally distinct isoforms of human CD63 were identified, one characteristic of vesicles and another expressed on the cell surface. Antibodies that differentiate between these two isoforms are therefore prospective diagnostic markers (Schäfer et al., 2010). Anti-CD63 mAb suppressed degranulation of adherent (but not non-adherent) RBL-2H3 cells, whereas synthesis of leukotrienes (LTs) was not affected. Initial stages of activation such as phosphorylation of signaling proteins and Ca^2+^ responses were not inhibited by the antibody. The ability of anti-CD63 mAb to suppress passive cutaneous anaphylaxis (PCA) *in vivo* makes CD63 a possible therapeutic target (Kraft et al., 2005). As mice deficient in CD63 have been recently prepared (Schröder et al., 2009), the role of CD63 for MC development and activation could be studied using this model. Interestingly, although CD63 is highly expressed in MCs, the number of MCs and their tissue distribution was not altered in CD63 KO mice, and also MCs derived from bone marrow of CD63 KO mice developed normally. When activated with Ag, but not phorbol-12-myristate-13-acetate/ionomycin, a significant decrease in FcεRI-mediated degranulation and tumor necrosis factor (TNF)-α secretion was observed in CD63-deficient BMMCs. On the other hand, secretion of IL-6 and LTC4 that are *de novo* synthetized upon activation was unaffected in BMMCs with CD63 KO. This finding, together with the fact that TNF-α is present in preformed granules, suggested that the absence of CD63 in the secretory granules is the main cause of reduced degranulation and TNF-α secretion (Kraft et al., 2013). To test the role of CD63 *in vivo*, the authors introduced CD63 wild-type (WT) and KO MCs into mice of MC-deficient strain *Kit*^w∕w−v^ and found out that mice reconstituted with CD63 KO MCs exhibited significantly decreased degranulation and PCA. CD63-specific antibody, unlike CD9-specific antibody, was unable to inhibit MC migration toward IL-16 (Qi et al., 2006).

## CD81

Similarly to antibodies directed toward CD63 (see above), mAb specific for CD81 was found to down-regulate FcεRI-mediated degranulation in RBL-2H3 cells without affecting initial tyrosine phosphorylation of signal-transduction proteins, calcium response and synthesis of LTC4. The inhibitory effect of anti-CD81 antibody was confirmed *in vivo*, where IgE-mediated PCA in rats was significantly decreased (Fleming et al., 1997). On the other hand, anti-CD81 antibody, unlike anti-CD9, was unable to inhibit MC migration toward IL-16 (Qi et al., 2006).

Despite the fact that production of CD81 KO mice was described almost 20 years ago (Maecker and Levy, 1997), the role of CD81 in MC development or activation has not been elucidated. A possible role of CD81 in MC activation was suggested based on experiments in which allergen-induced airway hyper-reactivity (AHR) was found to be diminished in CD81 KO mice. These mice, in contrast to WT mice, once challenged with ovalbumin (OVA) did not develop airway inflammation characterized by the presence of inflammatory cells, eosinophils, and synthesis of IL-4, IL-5, and IL-13 was also dramatically reduced. On the other hand, the serum levels of OVA-specific IgE were not changed (Deng et al., 2000). The authors speculated that the reduced levels of cytokines could be the reason of impaired MC activation in the absence of CD81 because anti-CD81 mAbs reduced MC activation. However, this idea has never been proved.

## CD151

Unlike other tetraspanins, CD151 was found to be upregulated after activation of FcεRI in human and mouse MCs. Using CD151 KO mice, Berdnikovs and colleagues studied the role of CD151 in MC physiology (Abdala-Valencia et al., 2015). The absence of CD151, did not lead to changes in degranulation after Ag activation. On the other hand, mice with CD151 KO exhibited a significant increase in magnitude of the late phase of PCA response (24–36 h). In line with these findings, CD151-deficient BMMCs showed enhanced production of several proinflammatory cytokines, including IL-4, IL-13, and TNF-α, which was probably related to the enhanced and sustained FcεRI-induced extracellular signal-regulated kinase (ERK)1/2 and protein kinase B (PKB, also called AKT) phosphorylation in these cells (Abdala-Valencia et al., 2015).

## CD53

Although CD53 is expressed in MCs, there is only one study showing association of CD53 with asthma risk via the functional promoter polymorphism. Interestingly, siRNA-mediated KD of CD53 in THP-1 human monocytic cells stimulated with house dust mite led to increased production of inflammatory cytokines as well as NFκB activity (Lee et al., 2013).

## Transmembrane adaptor proteins

Transmembrane adaptor proteins consist of a short extracellular domain, a single transmembrane domain and a long cytoplasmic tail. Extracellular domains are formed only by a few amino acids, and are therefore unlikely to function as receptors for extracellular ligands. The cytoplasmic tail possesses various tyrosine-containing motifs that could act, after phosphorylation, as scaffolds for anchor of multiple SRC homology (SH)2 domain-containing cytoplasmic as well as membrane-associated proteins and cytoskeletal components. As was already mentioned, most of the TRAPs possess a juxtamembrane palmitoylation motif that determines their solubility in non-ionic detergents, distribution in the plasma membrane, and some functional properties. The role of TRAPs in MC and leukocyte signaling has been recently extensively reviewed (Draber et al., 2011a, 2016; Horejsi and Hrdinka, 2014; Stepanek et al., 2014). Thus, this review is only limited to a short description of some structural properties of the most studied TRAPs in MCs that are important for the membrane organization (Table 2).

**Table 2:** **Selected TRAPs expressed in mast cells and their function**.

**Gene symbol**	**Most used aliases**	**KO**	**Phenotype of KO mice: general/in mast cells**
LAT	LAT1, pp36	Zhang et al., 1999	Defect in T-cell development—no mature T cells (Zhang et al., 1999)/reduced degranulation, Ca^2+^ release and cytokine production, PSA (Saitoh et al., 2000)
LAT2	LAB, NTAL, WBSCR15, WBSCR5	Volná et al., 2004; Zhu et al., 2004	Increased levels of natural antibodies and humoral response (Wang et al., 2005)/increased degranulation, Ca^2+^ release, cytokine production, PCA (Volná et al., 2004; Zhu et al., 2004), and chemotaxis (Tumová et al., 2010)
PAG1	PAG, CBP	Draberova et al., 2014	No visible changes in phenotype/reduced degranulation, Ca^2+^ release, cytokine production, chemotaxis, PCA (Draberova et al., 2014)
LAX1	LAX	Zhu et al., 2005a	Reduction in CD23 expression on mature B cells, spontaneous germinal center formation, hyper-responsiveness in T and B lymphocytes (Zhu et al., 2005a)/enhanced degranulation, cytokine production, cell survival (Zhu et al., 2006)
GAPT		Liu and Zhang, 2008	Increased B-cell proliferation and amount of Abs (Liu and Zhang, 2008)

## LAT

The cytoplasmic tail of LAT contains nine highly conserved tyrosine residues, of which five can be phosphorylated by spleen tyrosine kinase (SYK) kinase upon activation and serve as binding sites for SH2 domain-containing proteins including growth factor receptor-bound protein 2 (GRB2), phospholipase C (PLC)γ1, guanine nucleotide exchange factor VAV, ubiquitin ligase CBL, SH2 domain-containing leukocyte protein of 76 kDa (SLP-76), and GRB2-related adaptor downstream of SHC (GADS) (reviewed in Rivera, 2002, 2005; Draber et al., 2011a). Although development of MCs was not affected in mice deficient in LAT (Saitoh et al., 2000), the mice exhibited reduced Ag-mediated passive systemic anaphylaxis (PSA) responses. Ag-stimulated BMMCs from LAT-deficient mice showed reduced degranulation, Ca^2+^ release, and cytokine production (Saitoh et al., 2000), whereas their chemotaxis was unchanged (Hálová et al., 2013).

## NTAL

NTAL shares several structural features with LAT, including several tyrosine motifs of which five are the putative GRB2-binding sites. However, there is no major homology in amino acid sequences between these two adaptors and in contrast to LAT, NTAL lacks the PLCγ1 binding motif. Similarly to LAT, NTAL is rapidly phosphorylated upon Ag activation by SYK, and some tyrosines are also phosphorylated by LYN. In contrast to LAT, NTAL is also tyrosine phosphorylated upon c-KIT activation by stem cell factor (SCF), LYN, and c-KIT itself (Iwaki et al., 2008). Despite some similarities in features of LAT and NTAL structures, BMMCs isolated from NTAL KO mice exhibited increased degranulation, Ca^2+^ release, and cytokine production (Volná et al., 2004; Zhu et al., 2004). Chemotaxis toward Ag was also enhanced in NTAL-deficient cells (Tumová et al., 2010). In accord with these observations, PCA of NTAL-KO mice was also increased (Volná et al., 2004). Although it appeared that LAT and NTAL act as opposite regulators of MC signaling, it should be noted that the absence of both of them led to more extensive inhibition of FcεRI-induced activation than the absence of LAT alone (Volná et al., 2004; Zhu et al., 2004). Again, the only exception was chemotaxis that was increased in double KO cells compared to WT cells but still was lower than that in NTAL-deficient cells (Hálová et al., 2013). These data indicate that in the absence of NTAL, LAT acts as a negative regulator of chemotaxis.

## PAG

The structure of PAG is similar to that described for LAT and NTAL, but in addition to 10 tyrosines, it possesses two proline-rich domains that serve as binding sites for SH3 domains and a C-terminal VTRL motif for interaction with the PDZ domain of cytoskeletal linker ERM-binding protein of 50 kDa (EBP50) (Brdičková et al., 2001). Upon FcεRI triggering, PAG is phosphorylated by LYN in RBL-2H3 cells (Ohtake et al., 2002). Phosphorylated tyrosine 317 (human) or 314 (mouse) is crucial for binding of CSK. In RBL cells, overexpression of PAG led to inhibition of receptor phosphorylation and subsequent decreased degranulation (Ohtake et al., 2002). When BMMCs from mice with PAG KO were analyzed, different findings were obtained. PAG deficiency led to impaired Ag-induced degranulation, extracellular Ca^2+^ uptake, tyrosine phosphorylation of several proteins (including the FcεRI), production of cytokines and chemokines, and also decreased chemotaxis (Draberova et al., 2014). PAG-KO mice also exhibited impaired PCA. On the other hand, activation through c-KIT led to increased degranulation, suggesting different regulation of this TRAP after c-KIT and FcεRI activation (Draberova et al., 2014).

## Linker for activation of X cells (LAX)

LAX is another TRAP with multiple GRB2-binding motifs. However, in contrast to LAT and NTAL, LAX has no palmitoylation motif and is not localized in detergent-resistant membranes (DRMs) (Zhu et al., 2002). LAX-deficient MCs exhibited enhanced degranulation, enhanced activity of p38 mitogen-activated protein kinases (MAPK; PKB), and PI3K activation after stimulation via FcεRI. Cytokine production and cell survival were also enhanced in activated LAX-deficient cells. On the other hand, the absence of LAX had no effect on calcium response *in vitro* and PCA *in vivo* (Zhu et al., 2006).

## Grb2-binding adaptor protein, transmembrane (GAPT)

GAPT has a short extracellular domain, a transmembrane domain, and a cytoplasmic tail with multiple Grb2-binding motifs. Similarly to LAX, GAPT does not reside in DRMs, even though it contains potential palmitoylation sites similar to LAT and NTAL. On the other hand, palmitoylation of GAPT has not been proved. In contrast to other mentioned TRAPs, GAPT is not phosphorylated on tyrosine after FcεRI triggering (Liu and Zhang, 2008).

## Membrane micro/nano domains occupied by tetraspanins and TRAPs

Discovery of specific membrane domains came from the studies of insoluble residues that remained after lysis of the cells in buffers containing non-ionic detergents at 4°C. These DRMs formed predominantly by sphingolipids, cholesterol and proteins, were proposed to play key roles in membrane trafficking and signaling (Simons and Ikonen, 1997). Studies of DRMs were accelerated by development of the method of their isolation by sucrose density gradient centrifugation of cells lysed in non-ionic detergents. Both tetraspanins and palmitoylated TRAPs were found to be associated with DRMs. The effect of palmitoylation on the presence of these proteins in DRMs is discussed below. Based on the differences in detergent sensitivity, TEMs, or tetraspanin web, were identified as novel structures different from glycosylphosphatidylinositol (GPI)-microdomains and caveolae. It has been proposed that TEMs are involved in the plasma membrane organization through tetraspanin-tetraspanin and tetraspanin-other protein interaction (Boucheix and Rubinstein, 2001; Claas et al., 2001). In their sensitivity to different non-ionic detergents, protein-protein and protein-lipid interactions in TEMs differ from those in TRAP-enriched domains. Whereas tetraspanin-tetraspanin interactions are preserved in nonionic detergents Brij 58, Brij 97, Brij 98, and CHAPS, Triton X-100 disrupts the majority of these interactions along with participation of tetraspanins in sucrose low-density fractions. On the other hand, lysis in Brij detergents at 37°C disrupts GPI-microdomains but not TEMs (Claas et al., 2001; Charrin et al., 2003a, 2009). Tetraspanins were found to directly interact with cholesterol (Charrin et al., 2003b). Furthermore, it has been described that tetraspanins CD82 interacts with ganglioside GM2 (Todeschini et al., 2007), tetraspanin CD9 binds to GM3 (Kawakami et al., 2002) and that these interactions are important for the association of these tetraspanins with integrins. Since the concept of TEMs was proposed, most interactions of tetraspanins with their partners (e.g., tetraspanins and integrins) were identified by co-immunoprecipitation experiments after lysis of the cells in mild detergents. Some of them were later confirmed by crosslinking experiments or Förster resonance transfer (FRET) analysis (Boucheix and Rubinstein, 2001).

The existence of specific membrane domains that are dependent on lipid composition was at first deduced on the basis of experiments with detergent solubility/insolubility of individual membrane proteins and the term DRMs was coined. Discovery and use of modern high-resolution techniques, such as (FRET; McIntosh et al., 2012), fluorescence recovery after photobleaching (FRAP; Axelrod et al., 1976), stimulated emission depletion (STED; Auksorius et al., 2008), photoactivated localization microscopy (PALM; Betzig et al., 2006), and stochastic optical reconstruction microscopy (STORM; Rust et al., 2006) contributed significantly to identifying the real interactions on the plasma membrane components under *in vivo* conditions. The first results obtained with the high-resolution microscopic techniques (Kenworthy et al., 2000, 2004; Glebov and Nichols, 2004) challenged the theories based on detergent solubility. As more studies appeared, it was apparent that the majority of the results are basically in accord with the existence of plasma membrane domains, which are, however, of smaller size than previously thought, and therefore the term nanodomains was more often used for marking small dynamic domains that vary in time and size and which are enriched in cholesterol and sphingolipids.

Regarding tetraspanins, single-molecule fluorescence microscopy of living cells revealed that tetraspanin assemblies form dynamic interaction platforms in permanent exchange with the rest of the membrane. Tracking of tetraspanin CD9 showed that most of the time it was undergoing Brownian trajectories, but it was transiently trapped in platforms enriched in CD9 and its partners. Both the mobility and partitioning in the nanodomains were dependent on palmitoylation and plasma membrane cholesterol (Espenel et al., 2008). FRET-FLIM analysis revealed homophilic (CD9-CD9) and heterophilic (CD9-CD151) tetraspanin interactions as well as their interaction with adhesion molecules, with preferential association of CD9 with intercellular adhesion molecule 1 (ICAM-1, also known as CD54) and of CD151 with vascular cell adhesion molecule 1 (VCAM-1, also known as CD106). FRAP analysis also revealed that a marker of membrane microdomains, rGPI-EGFP, diffused much faster than tetraspanins (Barreiro et al., 2008; Ley and Zhang, 2008). On the other hand, recent work analyzed TEMs by STED microscopy and showed that tetraspanins form individual nanoclusters. It was also demonstrated that CD53 and CD37 domains showed only minor overlap with clusters containing tetraspanins CD81 or CD82. It should be also noted that CD53 and CD81 reside in closer proximity to their partners, major histocompatibility complex (MHC) class II and CD19, respectively, than to other tetraspanins (Zuidscherwoude et al., 2015).

When TRAPs were examined, using cells solubilized with non-ionic detergents, LAT was found in DRMs (Zhang et al., 1998). In contrast, FcεRI was not present in such domains before activation, but once aggregated, FcεRI became associated with DRMs. It has been suggested that association of FcεRI with DRMs is a prerequisite for FcεRI activation because in DRMs, FcεRI is phosphorylated by SRC family kinase LYN, which is resident in DRMs (Field et al., 1995; Dráberová et al., 2004). However, immunogold electron microscopy studies of isolated plasma membrane sheets did not prove co-localization of aggregated FcεRI and LAT, even though both domains were increased in size (Wilson et al., 2001, 2002; Lebduška et al., 2007). Scanning electron microscopy revealed that activation of RBL cells with Ag causes redistribution of LAT in the plasma membrane, where upon activation LAT was found in bigger clusters than before activation (Veatch et al., 2012). Although the studies did not examine whether LAT clusters co-localize with FcεRI clusters the data clearly showed that aggregation of FcεRIs causes redistribution of LAT. Recent studies of LAT clustering in resting and activated T cells by PALM (Betzig et al., 2006) and direct STORM (Rust et al., 2006) revealed that increase of LAT clusters after activation is due to the translocation of LAT from subsynaptic vesicles to cell surface and this recruitment is essential for LAT phosphorylation (Williamson et al., 2011). In another study with single and two-color PALM Sherman and collaborators showed that in resting and activated T cells LAT primarily resides in nanoscale clusters as small as dimers whose formation depended on protein-protein and protein-lipid interactions (Sherman et al., 2011). Furthermore, Lillemeier and collaborators used high-speed version of photoactivated localization microscopy (hsPALM), dual-color fluorescence cross-correlation spectroscopy (dsFCCS) and transmission electron microscopy and showed that both the T cell receptor (TCR) and LAT are preclustered into separate and spatially separated membrane domains on quiescent cells. After Ag recognition, these domains transiently concatenated into microclusters without any substantial change in the size and number of the component domains. These data suggest that partitioning immunoreceptors and their downstream signaling components into separate membrane domains, and then bringing these domains together, may be an important and general mechanism in the control of cell activation (Lillemeier et al., 2016). In another study, analysis of LAT in the plasma membrane of HeLa cells by fluorescence correlation spectroscopy (FCS) and PALM showed that LAT diffusion is retarded and its clustering in meso-scaled protein domains is decreased when associating with ordered-lipid domains in contrast to LAT associating with lipid-disordered domains (Owen et al., 2012). Importance of cholesterol for formation of DRMs and for immunoreceptor signaling has been repeatedly shown (Xavier et al., 1998; Sheets et al., 1999; Surviladze et al., 2001). It should also be noted that FcεRI-mediated activation is affected by ethanol, which seems to interfere with proper function of FcεRI-cholesterol signalosomes (Draberova et al., 2015) and that phosphatase inhibitor, pervanadate, induces FcεRI β and γ subunits tyrosine phosphorylation in the absence of FcεRI aggregation and its association with DRMs (Heneberg et al., 2010).

## Palmitoylation of tetraspanins and TRAPs

Palmitoylation is a reversible lipid post-translational modification of juxtamembrane cysteine residues, less frequently also serine and threonine, in a variety of transmembrane or membrane-associated proteins (Resh, 1999). Palmitoylation also allows such modified proteins to float in the low-density fraction of sucrose gradient after lysis in non-ionic detergents (Charrin et al., 2002, 2009; Stepanek et al., 2014). However, loss of palmitoylation of tetraspanins and TRAPs had a different impact on their solubility in non-ionic detergents and interactions with their partners. It has been shown that palmitoylation of CD151 had minimal influence on the density of tetraspanin-protein complexes and did not promote tetraspanin localization into DRMs or its association with α3β1 integrin, but its association with other cell surface proteins, including CD9 and CD63, was reduced (Yang et al., 2002). The palmitoylation of CD9 did not influence its localization into DRMs but was necessary for its interaction with other tetraspanins, namely CD81 and CD53 (Charrin et al., 2002). Similar observations were obtained when membrane compartmentalization of integrins had been studied. It has been suggested that palmitoylation of β4 promotes its association with DRMs and SRC family kinases (Gagnoux-Palacios et al., 2003) but further studies showed that β4 palmitoylation does not increase its localization into DRMs, instead it promotes CD151–α6β4 incorporation into a network of secondary tetraspanin interactions (Yang et al., 2004).

Although importance of palmitoylation for proper function of TRAPs has been shown, it still remains in part controversial. LAT is palmitoylated at C26 and C29 and this event is crucial for LAT association with DRMs and proper immunoreceptor function (Zhang et al., 1998; Levental et al., 2010). An important question was whether palmitoylation is also important for plasma membrane localization. It has been reported that the LAT mutated in the cysteines separately or together was localized into the plasma membrane of HEK cells (Zhang et al., 1998). However in this study LAT was not examined for its co-localization with plasma membrane markers. Recent studies showed that palmitoylation at C26 is essential for transporting of LAT from Golgi to plasma membrane (Hundt et al., 2009; Chum et al., 2016). In contrast, surprisingly, NTAL and PAG did not require palmitoylation for plasma membrane localization (Chum et al., 2016).

In another study, mutant LAT was constructed, in which transmembrane domain of LAT was exchanged with transmembrane domain of LAX, another TRAP, that lacks palmitoylation motifs, but possesses LAX transmembrane signal peptide that provides localization of the LAX in plasma membrane. This LAX-LAT protein was not detected in DRMs but appeared to be fully functional in T cell activation and development (Zhu et al., 2005b). Similar results were obtained when SRC-LAT mutant was constructed and examined in similar assays. The mutant protein was localized as peripheral membrane protein through myristoylation of its SRC domain but it was excluded from DRMs and appeared to be fully functional in TCR signaling (Hundt et al., 2009). Later studies of LAX-LAT mutant protein showed that it is localized in an atypical DRMs—called “heavy” DRMs (Otáhal et al., 2010). When CD25-LAT mutant was used, it was excluded from both DRMs and “heavy” DRMs (Otáhal et al., 2010). This study also demonstrated that the level of exogenous construct expression is critical for proper interpretation of the results. At the levels of expression, corresponding to the expression level of endogenous LAT, WT LAT, present mostly in DRMs, supported signaling better than LAX–LAT mutant; the CD25-LAT mutant was the least effective in the assay (Otáhal et al., 2010). Importance of palmitoylation for association with DRMs was also shown in studies with mutant LYN kinase in RBL cells. LYN mutated in both palmitoylation and myristoylation sites, did not anchor to the plasma membrane, whereas LYN with only palmitoylation site mutated was anchored to the plasma membrane, but its localization into DRMs was markedly reduced (Kovárová et al., 2001). Interestingly, studies with RBL cells showed that clustering of FcεRI led to co-clustering with LYN, depending on the presence of cholesterol (Veatch et al., 2012). Furthermore, FcεRI motility in the plasma membrane after Ag triggering was also dependent on cholesterol levels (Shelby et al., 2013).

## Tetraspanins and TRAPs cross-talk

Electron microscopy studies showed that LAT and NTAL, despite their structural similarities and proved association with DRMs, occupy separate membrane microdomains in MCs (Volná et al., 2004). The differences between the LAT and NTAL microdomains were confirmed in experiments in which topography of CD9 and TRAPs in the plasma membrane was examined: while NTAL co-localized with CD9, LAT did not (Hálová et al., 2013). CD9-NTAL co-localization was intensified by crosslinking CD9 with specific mAb, and this crosslinking resulted in enhanced phosphorylation of NTAL (Hálová et al., 2013). There are other examples of interaction between TRAPs and tetraspanins. Two recently identified TRAPs, SLP65/SLP76, Csk-interacting membrane protein (SCIMP) and leukocyte-specific transcript 1/A (LST1/A) were found to interact with tetraspanins (Draber et al., 2011b, 2012). SCIMP, which is expressed in B cells and other professional Ag-presenting cells, co-localized with tetraspanins CD81 and CD37 (Draber et al., 2011b), whereas LST1/A, which is expressed exclusively in the cells of myeloid origin (monocytes and granulocytes), co-localized with tetraspanins CD9 and CD81 (Draber et al., 2012).

Both tetraspanins and TRAPs were found to function as direct or indirect linkers of the plasma membrane and cytoskeleton. Co-immunoprecipitation studies showed that tetraspanins CD9 and CD81 are associated with ERM proteins, which directly interact with the cytoskeleton (Sala-Valdés et al., 2006). ERM proteins link plasma membrane phospholipids by binding their N-terminal FERM (4.1, ezrin/radixin/moesin) domain to phosphatidylinositol (4,5) bisphosphate (PIP_2_) and the actin cytoskeleton through the actin-binding domain (Abd) at the C-terminus. This binding is a highly dynamic process dependent on ERM 567/564/558 threonine phosphorylation/dephosphorylation. ERMs are released from the membrane when their inhibitory threonine is dephosporylated and ERMs are transformed from “open” phosphorylated to “closed” dephosphorylated conformation (McClatchey, 2014). However, it has not been clarified whether interactions between tetraspanins and ERMs are direct or indirect through tetraspanin-interacting proteins CD9P-1 and/or EWI-2 (Sala-Valdés et al., 2006). Earlier studies showed that engaging CD81 at the surface of B cells led to phosphorylation of ezrin by the SYK kinase (Coffey et al., 2009). In MCs, crosslinking of CD9 with mAb led to dephosphorylation of ERM inhibitory threonine (Hálová et al., 2013). TRAP PAG also interacts with ezrin, but in this case through ERM-binding phosphoprotein of 50 kDa (EBP50). In this binding, the N-terminal PDZ domain of EBP50 and C-terminal domain of PAG are involved (Brdičková et al., 2001). The ezrin-EBP50-PAG complex was found to be important for the spatio-temporal control of cAMP production through the CSK-PAG inhibitory pathway in effector T cells (Cornez and Taskén, 2010).

## Concluding remarks and perspectives

Although knowledge about tetraspanins and TRAPs at the level of proteins and the corresponding genes and their regulators is increasing, their functioning as platforms of plasma membrane signalosomes and their mutual crosstalk are far from understood. The exact composition of TEMs and TRAPs-enriched domains is also poorly defined. This is in part due to highly dynamic nature of the membrane domains and the large number of various lipids involved. Lipids function not only as a matrix in which tetraspanins, TRAPs, and other membrane proteins are anchored, but also as modifiers of various proteins. When focusing specifically on signal transduction in MCs, there are also many poorly explored areas, which include potential roles of various post-translational modifications on topography and function of the tetraspanins and TRAPs and their interaction partners. An important area to explore is the role of miRNAs, noncoding RNAs, and other regulators of tetraspanins and TRAPs expression (Zhang et al., 2014; Rouquette-Jazdanian et al., 2015). As shown in this review focused on MCs, important discoveries concerning the role of individual members of tetraspanins and TRAPs were brought by studies based on cells deficient in selected proteins through the gene KO or KD approach. However, with the armamentarium of proteomics, lipidomics, epigenetics, super-resolution microscopy, multi-photon microscopy, etc., our pace of discoveries in the field of specific membrane nanodomains will be accelerated in the next few years. Most of the studies mentioned in this review were performed using cells cultured under *in vitro* conditions. However, one needs to understand more on how TEMs and TRAPs-enriched domains function under *in vivo* conditions. This issue involves not only the influence of various soluble factors that are absent in cell culture media, but also the interaction with other cells and extracellular matrix components that could influence functions of the tetraspanins and their interaction partners. Such type of studies in the case of MCs will deepen our understanding of the cellular and molecular mechanisms underlying allergic diseases.

## Author contributions

Both authors have made substantial, direct and intellectual contribution to the work, and approved it for publication.

### Conflict of interest statement

The authors declare that the research was conducted in the absence of any commercial or financial relationships that could be construed as a potential conflict of interest.
